# Infiltration of Autologous Growth Factors in Chronic Tendinopathies

**DOI:** 10.1155/2015/924380

**Published:** 2015-06-15

**Authors:** Antonio Crescibene, Marcello Napolitano, Raffaella Sbano, Enrico Costabile, Hesham Almolla

**Affiliations:** ^1^Orthopedics and Traumatology Unit, San Francesco di Paola Hospital, Via Promintesta, 87027 Paola, Italy; ^2^Immunohaematology and Transfusion Medicine Unit, Cosenza Hospital, Via Migliori, 87100 Cosenza, Italy; ^3^Radiology Unit, San Francesco di Paola Hospital, Via Promintesta, 87027 Paola, Italy; ^4^Orthopedics and Traumatology Unit, Cosenza Hospital, Via Migliori, 87100 Cosenza, Italy; ^5^Radiology Unit, Cosenza Hospital, Via Migliori, 87100 Cosenza, Italy

## Abstract

Achilles tendinopathy and patellar tendinopathy are among the most frequent diagnoses in sports medicine. Therapeutic treatment of the disease is difficult, particularly in chronic cases. In literature, several studies suggest the employment of Platelet-Rich Plasma as a therapeutic alternative in tendinopathies. The choice of employing this method is based on the activity of growth factors contained in platelets which activate, amplify, and optimize the healing process. We selected 14 patients affected by Achilles tendinopathy and 7 patients affected by patellar tendinopathy, with a two-year final follow-up. These patients underwent a cycle of three tendinous infiltrations, after clinical and instrumental evaluation carried out by means of specific questionnaires and repeated ultrasound scans. Ultrasound scans of 18 patients showed signs of reduction in insertional irregularities. The result is confirmed by complete functional recovery of the patients, with painful symptomatology disappearing. The patients showed a clear pain reduction, along with an enhanced VISA score after the 24-month follow-up, equal to 84.2 points on a scale of 0 to 100. In conclusion, the present study provides evidence to suggest that PRP infiltration is a valid option to patients with chronic tendinopathy who did not benefit from other treatments.

## 1. Introduction

In the last three decades, a considerable increase in the incidence of excess functional load pathologies has been reported. Not only did this occur because of the growing number of amateur sportspersons, but this also occurred because of lengthier and tougher trainings and sport events among professionals. Repeated traumas and microtraumas have been identified as the major cause of such pathologies, which affect those practising basketball, volleyball, tennis, skiing, and soccer [1, 2].

Achilles tendon inflammation was predominantly present in 11% to 24% of joggers, whereas patellar tendinopathy was found in 32% to 45% of basketball and volleyball players [3].

In nonresponsive conditions, before deciding on a surgical solution, it is worth considering an eco-guided PRP intratendinous infiltration.

Tendon tissue recovery is rather long, and this limited capacity of recovery is supposed to be caused by poor tendinous vascularisation [4].

In the region of the lesion, platelet degranulation occurs; this releases several growth factors such as TGF (Transforming Growth Factor), PDGF (Platelet Derived Growth Factor), EGF (Epidermal Growth Factor), VEGF (Vascular Endothelial Growth Factor), and IGF (Insulin-Like Growth Factor).

The first three, in particular, encourage cellular proliferation and migration through protein synthesis in the extracellular matrix.

Some other cytokines are released, such as HGF (Hepatocyte Growth Factor) and bFGF (Basic Fibroblast Growth Factor); they are chemotactic and mitogenic to endothelial cells and encourage angiogenesis and revascularization, two fundamental processes in tissue regeneration [5].

In literature, several studies suggest the employment of PRP as a successful therapeutic alternative in tendinopathies [6–8] because growth factors activate, amplify, and optimize the healing process [9–11].

## 2. Materials and Methods

We selected 21 patients affected by chronic tendinopathy, with persisting symptomatology for at least six months. Between September 2010 and March 2012, they underwent treatment of PRP infiltration.

The first stage involved visit, medical history reconstruction, and evaluation of the hemochromocytometric profile of the outpatients.

We considered as discriminating factors hemoglobin values <11 g/dL, a number of white blood cells >10 × 10^Λ3^/*μ*L, and a number of platelets <120 × 10^Λ3^/*μ*L.

All patient presented value in range of normality (Tables 1 and 2).

The inclusion criteria for the study were the presence of patellar or Achilles tendinopathy, pain at palpation and during physical activity for at least four months, insertional irregularity in ultrasound scanning [7].

All patients had previously undergone other treatments: intake of NSAIDs, rehabilitation programme, laser therapy, corticosteroids injections, with unsatisfactory outcome.

We also excluded patients with systemic pathologies such as diabetes mellitus, cancer, severe cardiovascular pathologies, immunodepression, anticoagulating, or antiaggregating therapy [7].

These conditions do not allow the application of the technique in the limits of safety for the patient that may develop infective complications and adverse effects.

The patients (14 men and 7 women) had an average age of 40, ranging from 16 to 70.

The average Body Mass Index (BMI) was 25, ranging from 23 to 26. In 13 cases, the left-hand side of the body was affected, with the remaining 8 patients affected in their right-hand side; the patients' blood tests were all normal, with no contraindications.

We treated 14 cases of Achilles tendinopathy and 7 cases of patellar tendinopathy.

All of the patients were sportspersons; there were 10 professionals among them. They underwent a cycle of three infiltrations, one per week.

During the initial pretreatment evaluation we administered some suitable questionnaires: the Numeric Rating Scale (NRS) [12] for measuring subjective pain, as well as the Victorian Institute of Sport Assessment-Achilles (VISA-A) questionnaire [13] and the Victorian Institute of Sport Assessment-Patellar (VISA-P) questionnaire [14], designed for Achilles and patellar tendinopathies, respectively.

These evaluations were repeated at the end of the cycle and throughout the follow-up.

The VISA score measures the ability to practise sports activities by means of a 0–100 score, in which the highest score represents full activity.

All patients read and signed informed consent and the paper was performed in accordance with the Ethical standards of the 1964 Declaration of Helsinki as revised in 2000.

All procedures were performed in accordance with the ethical standards of the institutional and/or national research committee.

For statistical analyses we used the Mann-Whitney *U* or Wilcoxon Rank-Sum Test for Difference in Medians (*t*-test two samples).

**Figure 1:**
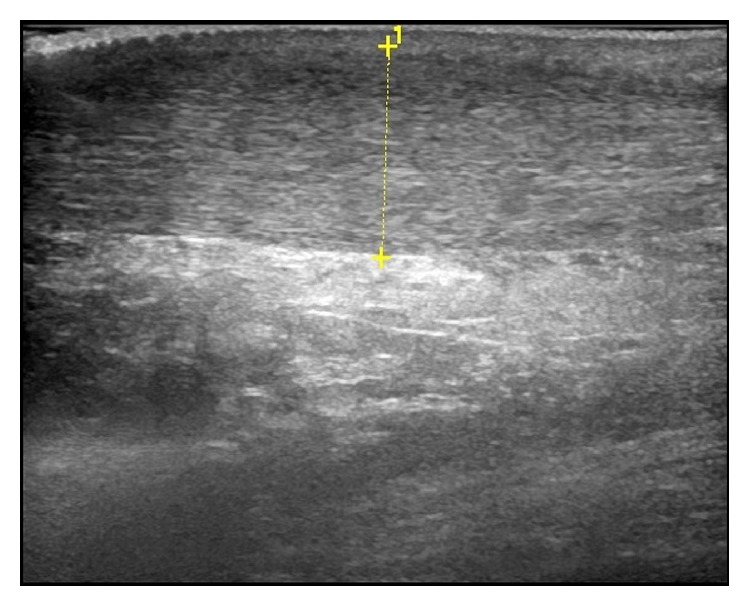
Pretreatment ultrasound image of the Achilles tendon.

In order to reduce intrinsic variables in ultrasound scanning, we decided on making use of the usual X-ray specialist and a* Logiq E9* scanner (Figure 1) with a 4 MHz multifrequency linear probe.

Any kind of sports activity was forbidden during this therapy. The patients followed a personalized therapeutic program consisting of an initial stage with cautious active mobilization of the region and hydrokinesitherapy, followed by eccentric exercises with constant check from the physiotherapist, for one month.

At the end of this program, patients could gradually resume their usual sports activities, by encouraging patients to perform stretching and eccentric exercises, in an average period of six weeks.

All the procedures were performed under control of the Chief Immunohematologist at the Hospital Department of Immunohematology and Transfusional Medicine, in accordance with Decree number 191/2005 [15], with prior acquisition of informed consent.

Each infiltration session encompassed a preparation stage. We collected a venous blood sample of about 8 cc into a RegenLab Fibrin Polymer 2 test tube and then centrifuged it at 3100 rpm for eight minutes.

By doing so, we obtained the separation through physical principles of erythrocytes from plasma, while platelets sedimented on the surface of separation [10].

We obtained in this way the PRP, to which we added 10% calcium gluconate [16], and then immediately apply the substance through infiltration; the gelification process occurred within 2–7 minutes due to body heat [17].

We use 1 cc of PRP in order to carry out quality controls and calculate the number of platelets in PRP.

We registered plasmatic platelet concentration of 276.8 × 10^Λ3^ *μ*L, platelet recovery of 25.3%, and a concentration factor of 2.2.

After careful disinfection of the infiltration site with didecyldimethylammonium chloride, we anesthetized the cutaneous and subcutaneous tissue with 2 cc of 2% lidocaine and then inserted a needle 22 gauge into the tendon, with ultrasound scanning as guidance.

**Figure 2:**
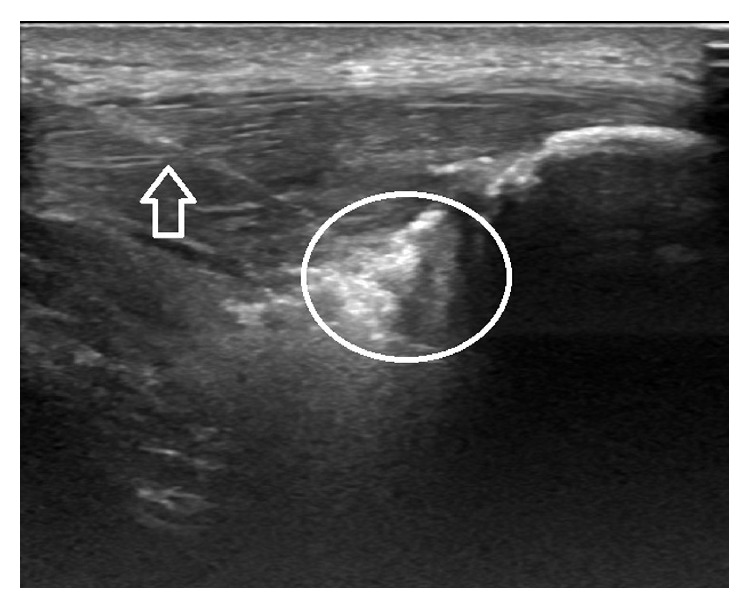
The arrow indicates the needle and the circle shows the PRP infiltration point in a case of patellar tendinopathy.

We now injected about half of the PRP into the tendon and the rest into the peritendinous area (Figure 2).

We removed the needle, disinfected the region, and applied a dressing. We left the limb to rest for fifteen minutes.

Throughout treatment we forbade patients to take nonsteroidal anti-inflammatory drugs and suggested avoiding for at least two weeks intense sports activities which might involve use of the legs.

## 3. Results

18 patients completed the 24-month final follow-up, while 3 interrupted periodical visits after 12 months (one patient with Achilles tendinopathy and two affected by patellar tendinopathy).

The NRS scale for measuring subjective pain registered an average initial score of 6.6 ± 1.0 and a posttreatment average score of 1.2 ± 1.2 (*P* < 0.01).

We observed a VISA score enhancement, with an average pretreatment score of 46.4 ± 18.7 and an average posttreatment one of 82 ± 12.4.

The follow-up indicated an average score of 85.2 ± 3.0 after 12 months and an average one of 84.2 ± 2.9 after 24 months (*P* < 0.01).

We noticed a significant improvement in patients with Achilles tendinopathy, whose VISA-A questionnaires showed, at each of the evaluation stages previously mentioned, the following results: 52.6 ± 20.1, 88.8 ± 3.4, 86.2 ± 3.1, and 85.2 ± 3.2 (*P* < 0.01).

The NRS scale for measuring subjective pain registered, in this group, a pretreatment average value of 6 ± 1 and a posttreatment average value of 0.6 ± 0.8 (*P* < 0.01) (Table 3).

We observed a less remarkable improvement in patients affected by patellar tendinopathy, whose VISA-P questionnaires suggested, at each of the evaluation stages, the following average scores: 40.2 ± 17.0, 75.2 ± 14.7, 84 ± 2.9, and 83 ± 2.4 (*P* < 0.01).

The NRS scale for assessment of pain intensity revealed, in this group, average results of 7.2 ± 0.8 and 1.8 ± 1.3 (*P* < 0.01) (Table 4).

Patients with Achilles tendinopathy showed the best scores of the study population and they accounted for 72% of the population studied.

**Figure 3:**
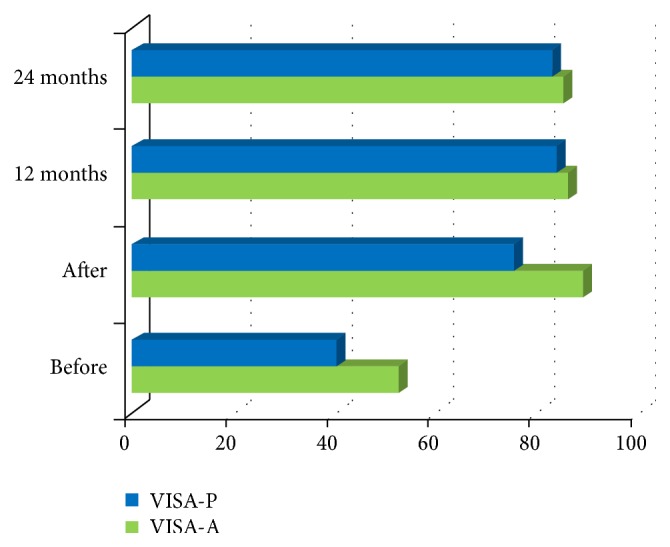
VISA evaluation of patellar and Achilles tendinopathy.

We have noticed a score lowest in the population suffering from patellar tendinopathy; nevertheless, the patients have taken the sport with good final scores (Figure 3).

At the end of the follow-up, ultrasound scans of the 18 patients showed signs of reduction in insertional irregularities. This result is clinically confirmed by complete functional recovery of the patients, with painful symptomatology disappearing.

Moreover, a good tolerance to the use of PRP was observed, with the absence of any adverse effects related to such therapy.

## 4. Discussion

Achilles tendinopathy and patellar tendinopathy are among the most frequent diagnoses in sports medicine. Therapeutic treatment of the disease is difficult, particularly in chronic cases.

The most reliable and secure treatment is still the conservative recovery program, which encompasses eccentric specific and gradual muscular work, although the percentage of positive results in extreme chronic tendinopathies is about 50% [18, 19].

Since tendon tissue is avascularized, the healing process is bound to be disrupted and tendinopathies are bound to become chronic.

Tendon recovery with formation of scar tissue involves loss of the mechanical properties of elasticity and resistance which are typical of healthy tendon tissue.

The healing process takes place in three main phases: inflammatory phase, proliferative phase and remodeling.

In the first phase, platelets play the physiological role of starting and modulating the healing process.

Recent studies have proved that use of autologous growth factors might be helpful in a number of conditions.

The effect of individual growth factors in models of in vivo and in vitro tendon repair has already been described in literature [6–8, 20–22].

Deans et al. [6] obtained statistically remarkable results in terms of pain, various other symptoms, everyday activities, sports activities, and quality of life in a group of 26 patients with chronic Achilles tendinopathy. Ferrero et al. [7] reported a significant and long-term improvement in clinical symptomatology, accompanied by recovery of tendon matrix, in a group of 48 patients affected by chronic Achilles and patellar tendinopathies. Charousset et al. examined 28 athletes with chronic patellar tendinopathy refractory to nonoperative management. In this study, application of 3 consecutive US-guided PRP injections significantly improved symptoms and function in athletes with chronic patellar tendinopathy and allowed fast recovery to their presymptom sporting level [8].

Research by Sánchez et al. [23] demonstrated better results in a group of patients whose Achilles tendon was repaired through PRP application; they used a control group to compare results.

The present study on the use of PRP to treat chronic tendinopathies has been prompted by the safe autologous nature of this therapy, its mechanism of action based on basic biological principles, and promising works in scientific literature.

The group of patients we examined was regarded as a good model in evaluating the benefits of PRP application in chronic tendinopathies, but further randomized controlled studies performed on a larger sample size are warranted to confirm these preliminary results.

The limitations of this study are the small number of patients and the lack of control group.

This process was integrated with a rehabilitation program and patients' progress was measured by means of the VISA score and evaluation of serialized ultrasound images.

## 5. Conclusions

The patients showed a clear pain reduction, along with a VISA score after the 24-month follow-up equal to 84.2 points on a scale of 0 to 100.

**Figure 4:**
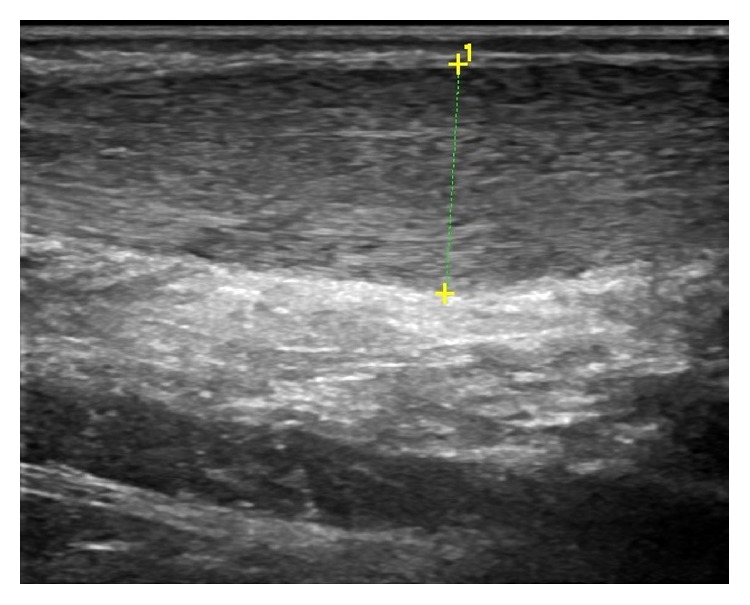
Ultrasound scan control of the Achilles tendon on 24-month follow-up.

From ultrasound scans, we noticed a visible reduction in tissue irregularity in 86% of infiltrated tendons (Figure 4).

In conclusion, the present study provides evidence to suggest that PRP infiltration is a valid option to patients with chronic tendinopathy who did not benefit from other treatments.

## Figures and Tables

**Table 1:** Laboratory values of Achilles tendinopathy.

Patient	RBC 10^6^/*μ*L	HGB g/dL	HCT %	PLT 10^3^/*μ*L	WBC 10^3^/*μ*L
SL	5.15	13.8	43.6	236	9.49
MF	4.91	12.9	40.7	336	9.2
GG	3.85	12.5	36.3	321	6.21
SP	5.02	15.4	46.1	201	5.5
MML	5.90	11.7	37.5	301	8.0
MF	4.81	14.7	40.2	218	5.36
BR	5.23	15.0	44.2	204	4.0
NP	5.17	15.2	44.5	252	8.1
BM	4.21	13.6	38.2	264	7.55
CG	3.67	12.0	35.1	304	7.2
CD	5.52	16.0	44.2	215	6.14
IGA	4.88	14.5	43.3	261	7.58
PG	5.06	15.5	43.1	174	5.35
CR	4.73	13.1	38.7	284	8.6

RBC: red blood cells; HGB: hemoglobin; HCT: hematocrit; PLT: platelets; WBC: white blood cells.

**Table 2:** Laboratory values of patellar tendinopathy.

Patient	RBC 10^6^/*μ*L	HGB g/dL	HCT %	PLT 10^3^/*μ*L	WBC 10^3^/*μ*L
CM	4.08	12.3	37.1	253	5.21
BG	4.32	13.6	39.0	197	5.07
SD	5.81	16.0	47.9	243	4.74
CG	5.27	15.1	44.5	278	6.54
ME	4.45	13.9	40.6	183	6.05
OR	5.12	14.8	44.2	227	6.0
DZ	4.75	13.9	41.0	265	6.0

**Table 3:** Clinical evaluation of Achilles tendinopathy (values are presented as mean).

Patient	NRS before	NRS after	VISA-A before	VISA-A after	VISA-A F.U. 12 months	VISA-A F.U. 24 months
SL	5	0	31	90	82	80
MF	7	1	33	84	89	81
GG	6	2	73	93	91	85
SP	5	0	69	87	87	86
MML	7	1	70	92	85	88
MF	8	2	35	85	81	89
BR	5	0	85	89	88	84
NP	6	1	46	86	87	80
BM	5	0	32	94	88	89
CG	6	0	64	92	89	88
CD	5	0	62	90	89	88
IGA	5	0	22	83	81	—
PG	7	2	43	89	85	85
CR	7	0	72	90	86	85

**Table 4:** Clinical evaluation of Patellar Tendinopathy (Values are presented as mean).

Patient	NRS before	NRS after	VISA-P before	VISA-P after	VISA-P F.U. 12 Months	VISA-P F.U. 24 Months
CM	7	2	23	56	81	—
BG	8	4	55	85	86	83
SD	7	0	17	90	88	86
CG	8	3	27	53	80	—
ME	6	2	52	82	85	83
OR	8	1	54	84	82	83
DZ	7	1	54	77	86	80

## References

[1] Alfredson H., Pietilä T., Jonsson P., Lorentzon R. (1998). Heavy-load eccentric calf muscle training for the treatment of chronic achilles tendinosis. *American Journal of Sports Medicine*.

[2] Yu J. S., Popp J. E., Kaeding C. C., Lucas J. (1995). Correlation of MR imaging and pathologic findings in athletes undergoing surgery for chronic patellar tendinitis. *American Journal of Roentgenology*.

[3] Wasielewski N. J., Kotsko K. M. (2007). Does eccentric exercise reduce pain and improve strength in physically active adults with symptomatic lower extremity tendinosis? A systematic review. *Journal of Athletic Training*.

[4] Nirschl R. P., Ashman E. S. (2003). Elbow tendinopathy: tennis elbow. *Clinics in Sports Medicine*.

[5] Marx R. E., Carlson E. R., Eichstaedt R. M., Schimmele S. R., Strauss J. E., Georgeff K. R. (1998). Platelet-rich plasma: growth factor enhancement for bone grafts. *Oral Surgery, Oral Medicine, Oral Pathology, Oral Radiology, and Endodontics*.

[6] Deans V. M., Miller A., Ramos J. (2012). A prospective series of patients with chronic Achilles tendinopathy treated with autologous-conditioned plasma injections combined with exercise and therapeutic ultrasonography. *Journal of Foot and Ankle Surgery*.

[7] Ferrero G., Fabbro E., Orlandi D. (2012). Ultrasound-guided injection of platelet-rich plasma in chronic Achilles and patellar tendinopathy. *Journal of Ultrasound*.

[8] Charousset C., Zaoui A., Bellaiche L., Bouyer B. (2014). Are multiple platelet-rich plasma injections useful for treatment of chronic patellar tendinopathy in athletes? a prospective study. *American Journal of Sports Medicine*.

[9] Nguyen R. T., Borg-Stein J., McInnis K. (2011). Applications of platelet-rich plasma in musculoskeletal and sports medicine: an evidence-based approach. *PM & R*.

[10] Dohan Ehrenfest D. M., Andia I., Zumstein M. A., Zhang C.-Q., Pinto N. R., Bielecki T. (2014). Classification of platelet concentrates (Platelet-Rich Plasma-PRP, platelet-rich fibrin-PRF) for topical and infiltrative use in orthopedic and sports medicine: current consensus, clinical implications and perspectives. *Muscles, Ligaments and Tendons Journal*.

[11] Dallaudière B., Pesquer L., Meyer P. (2014). Intratendinous injection of platelet-rich plasma under US guidance to treat tendinopathy: a long-term pilot study. *Journal of Vascular and Interventional Radiology*.

[12] Jensen M. P., Karoly P., Braver S. (1986). The measurement of clinical pain intensity: a comparison of six methods. *Pain*.

[13] Robinson J. M., Cook J. L., Purdam C. (2001). The VISA-A questionnaire: a valid and reliable index of the clinical severity of Achilles tendinopathy. *British Journal of Sports Medicine*.

[14] Zwerver J., Kramer T., van den Akker-Scheek I. (2009). Validity and reliability of the Dutch translation of the VISA-P questionnaire for patellar tendinopathy. *BMC Musculoskeletal Disorders*.

[16] Sánchez M., Guadilla J., Fiz N., Andia I. (2012). Ultrasound-guided platelet-rich plasma injections for the treatment of osteoarthritis of the hip. *Rheumatology*.

[17] Zimmermann R., Jakubietz R., Jakubietz M. (2001). Different preparation methods to obtain platelet components as a source of growth factors for local application. *Transfusion*.

[18] Childress M. A., Beutler A. (2013). Management of chronic tendon injuries. *American Family Physician*.

[19] Woodley B. L., Newsham-West R. J., Baxter G. D. (2007). Chronic tendinopathy: effectiveness of eccentric exercise. *British Journal of Sports Medicine*.

[20] Anitua E., Andía I., Sanchez M. (2005). Autologous preparations rich in growth factors promote proliferation and induce VEGF and HGF production by human tendon cells in culture. *Journal of Orthopaedic Research*.

[21] Klein M. B., Yalamanchi N., Pham H., Longaker M. T., Chang J. (2002). Flexor tendon healing in vitro: effects of TGF-*β* on tendon cell collagen production. *Journal of Hand Surgery*.

[22] Natsu-ume T., Nakamura N., Shino K., Toritsuka Y., Horibe S., Ochi T. (1997). Temporal and spatial expression of transforming growth factor-*β* in the healing patellar ligament of the rat. *Journal of Orthopaedic Research*.

[23] Sánchez M., Anitua E., Azofra J., Andía I., Padilla S., Mujika I. (2007). Comparison of surgically repaired Achilles tendon tears using platelet-rich fibrin matrices. *The American Journal of Sports Medicine*.

